# Supplementary material for: Mapping the evolution of fertility support policies in China: A content and instrumental analysis
Source: PLoS One. 2025 Oct 9;20(10):e0332137. doi: 10.1371/journal.pone.0332137 (PMC12510515; doi:10.1371/journal.pone.0332137)
Supplement: S1 Appendix — (ZIP) [file pone.0332137.s001.zip › S1 Appendix. 226 original policy documents/60-国务院办公厅关于印发全国深化“放管服”改革优化营商环境电视电话会议重点任务分工方案的通知(FBM-CLI-2-334917).doc]

国务院办公厅关于印发全国深化“放管服”改革优化营商环境电视电话会议重点任务分工方案的通知

**发布部门：** [国务院办公厅](https://www.pkulaw.com/law/chl?Aggs.IssueDepartment=601) [机构沿革](javascript:void(0))

**发文字号：**国办发〔2019〕39号

**发布日期：**2019.08.01

**实施日期：**2019.08.01

**时效性：** [现行有效](https://www.pkulaw.com/law/chl?Aggs.TimelinessDic=01)

**效力级别：** [国务院规范性文件](https://www.pkulaw.com/law/chl?Aggs.EffectivenessDic=XC0203)

**法规类别：** [市场管理](https://www.pkulaw.com/law/chl?Aggs.Category=08204) [营商环境优化](https://dobusiness.pkulaw.com/)

国务院办公厅关于印发全国深化“放管服”改革优化营商环境电视电话会议重点任务分工方案的通知
（国办发〔2019〕39号）

各省、自治区、直辖市人民政府，国务院各部委、各直属机构：
　　《全国深化“放管服”改革优化营商环境电视电话会议重点任务分工方案》已经国务院同意，现印发给你们，请结合实际认真贯彻落实。

国务院办公厅
2019年8月1日

　　全国深化“放管服”改革优化营商环境电视电话会议重点任务分工方案

　　党中央、国务院高度重视深化“放管服”改革、优化营商环境工作。2019年6月25日，李克强总理在全国深化“放管服”改革优化营商环境电视电话会议上发表重要讲话，部署深化“放管服”改革，加快打造市场化法治化国际化营商环境。为确保会议确定的重点任务落到实处，现制定如下分工方案。
　　**一、**推动简政放权向纵深发展，进一步放出活力
　　（一）进一步放宽市场准入，缩减[市场准入负面清单](https://www.pkulaw.com/chl/d8135b05437494cebdfb.html)事项，清单之外不得另设门槛和隐性限制。（发展改革委、商务部牵头，国务院相关部门及各地区按职责分工负责）
　　具体措施：
　　1.2019年9月底前修订形成新版[市场准入负面清单](https://www.pkulaw.com/chl/d8135b05437494cebdfb.html)，缩减清单事项，以服务业为重点试点进一步放宽市场准入限制。推行“全国一张清单”管理模式，建立全国统一的清单代码体系，严禁各地区、各部门自行发布市场准入性质的负面清单。（发展改革委、商务部负责）
　　2.2019年修订《[公平竞争审查制度实施细则（暂行）](https://www.pkulaw.com/chl/1dcf6052cfdd94c0bdfb.html)》，组织各地区、各部门完成存量文件专项清理，修改、废止一批不利于公平竞争的政策措施，加快研究制定实施竞争政策的指导意见。（市场监管总局牵头，各地区、各部门负责）
　　3.2019年9月底前部署开展招投标领域专项整治，全面清理各级政府及有关部门规章、行政规范性文件和其他政策措施中对民营、外资企业投标设置不合理限制和壁垒的规定，纠正并查处一批不合理限制或排斥潜在投标人等违法违规行为。（发展改革委等国务院相关部门按职责分工负责）
　　4.加强对各地区、各部门政府采购活动的指导和监管，清理政府采购领域妨碍统一市场与公平竞争的规定和做法。（财政部、市场监管总局负责）
　　（二）继续压减中央和地方层面设定的行政许可事项。2019年内中央层面再取消下放一批行政许可事项。对保留的许可事项要逐项明确许可范围、条件和环节等，能简化的都要尽量简化。（国务院办公厅牵头，各地区、各部门负责）
　　具体措施：
　　1.2019年底前研究提出50项以上拟取消下放和改变管理方式的行政许可事项，清理简并多部门、多层级实施的重复审批。编制公布中央层面设定的行政许可事项清单，逐项简化并明确许可范围、许可条件、许可有效期限等，细化审批标准、办理程序和时限等要求。（国务院办公厅牵头，国务院相关部门按职责分工负责）
　　2.组织清理规范地方层面设定的行政许可事项，2019年底前以省为单位集中统一公布地方层面设定的行政许可事项清单。（国务院办公厅牵头，各地区负责）
　　3.深化交通运输领域简政放权。简化道路客运经营许可事项和申请材料，精简道路货运从业驾驶员培训考试。优化机动车产品准入，依法整合汽车产品公告、强制性产品认证、环保型式核准目录、道路运输车辆燃料消耗量达标车型公告等，实现一次送检、全面检测、结果互认。2019年底前形成货车“三检合一”实施方案。（发展改革委、公安部、交通运输部、生态环境部、工业和信息化部、市场监管总局按职责分工负责）
　　（三）继续压减工业产品生产许可证，2019年把许可证种类再压减一半以上。（市场监管总局负责）
　　具体措施：
　　1.进一步调整工业产品生产许可证管理目录，2019年9月底前将发证产品种类从24类压减至12类以内，压缩发证层级，减少产品单元。对保留和退出目录的产品都要制定加强事中事后监管的措施。（市场监管总局负责）
　　2.2019年10月底前将强制性产品认证目录再压减30种以上，继续扩大“自我声明”评价方式实施范围，简化强制性认证管理程序，减少认证证书种类数量。（市场监管总局负责）
　　3.推动检验检测认证机构与政府部门彻底脱钩，鼓励社会资本进入检验检测认证市场。清理检验检测行政许可，实施统一的资质认定管理，加快实现检验检测认证结果在不同部门、层级和地区间互认通用，避免重复评价。（市场监管总局牵头，国务院相关部门按职责分工负责）
　　（四）大力清理简并种类过多、划分过细的资质资格许可事项。（人力资源社会保障部、自然资源部、住房城乡建设部、交通运输部等国务院相关部门按职责分工负责）
　　具体措施：
　　1.推动技能人员水平评价类职业资格分批调整退出国家职业资格目录，2019年底前建立完善职业技能等级制度，推动实现由用人单位和第三方机构开展职业技能等级认定、颁发职业技能等级证书，政府不再颁发职业技能等级证书。对涉及国家安全、公共安全、生态环境安全、人身健康、生命财产安全的水平评价类职业资格，确需实施准入管理的，依照法定程序调整为准入类职业资格。2020年底前将国家职业资格数量再压减一半以上。（人力资源社会保障部牵头，国务院相关部门按职责分工负责）
　　2.大幅压减企业资质资格认定事项，力争2020年底前将工程建设、测绘等领域企业资质类别、等级压减三分之一以上，凡是能由市场机制调节的一律取消，对保留的事项要精简资质类别、归并等级设置。探索对部分资质资格实行告知承诺管理。（自然资源部、住房城乡建设部、交通运输部、应急部等国务院相关部门按职责分工负责）
　　（五）整治各类变相审批，摸清备案、登记、年检、认定等部门管理措施的底数，并持续清理压减。对确需保留的实行清单管理，并向社会公开。（国务院办公厅牵头，各地区、各部门负责）
　　具体措施：
　　2019年底前启动清理规范中央层面规定的目录、备案、计划、规划、登记、注册、年检、年报、监制、认定、认证、审定等管理措施，依法依规取消变相审批，分类编制事项清单并明确办理规则和流程。2020年6月底前研究提出一批简化、优化的管理措施，改为通过信息共享等方式优化办事流程。（国务院办公厅牵头，国务院相关部门按职责分工负责）
　　（六）继续推进“证照分离”改革，重点是“照后减证”。（国务院办公厅、市场监管总局、司法部牵头，国务院相关部门按职责分工负责）
　　具体措施：
　　2019年底前在自由贸易试验区启动“证照分离”改革全覆盖试点工作，将中央层面和地方层面设定的涉企经营许可事项全部纳入改革范围，通过直接取消审批、审批改为备案、实行告知承诺、优化审批服务等四种方式分类推进改革，2020年下半年在全国推开。（国务院办公厅、市场监管总局、司法部牵头，相关地区及国务院相关部门按职责分工负责）
　　（七）进一步压减企业开办时间，2019年底前压减到5个工作日以内，有条件的地方要压减到3个工作日以内。（市场监管总局牵头，国务院相关部门及各地区按职责分工负责）
　　具体措施：
　　1.加快电子营业执照、电子印章推广应用，简并现场登记环节。推广企业开办全程网上办，鼓励具备条件的地区加快实现开办企业时申领营业执照、刻制印章、申领发票、申领税控设备、社保登记、住房公积金开户登记等全流程网上申请和办理，现场“一窗”、一次领取企业开办全部材料。（市场监管总局牵头，公安部、住房城乡建设部、人力资源社会保障部、税务总局等国务院相关部门及各地区按职责分工负责）
　　2.2019年底前建成全国统一的电子发票公共服务平台，为纳税人提供免费的电子发票开具服务，加快电子发票的推广应用。尽快研究推进增值税专用发票电子化。2019年底前将纳税人办税事项、纳税时间再压减10%，70%以上办税事项实现一次办结。（税务总局、财政部按职责分工负责）
　　（八）进一步完善市场主体退出机制，促进优胜劣汰。（发展改革委牵头，国务院相关部门及各地区按职责分工负责）
　　具体措施：
　　1.2019年8月底前推动各地建成企业注销网上服务专区，实现企业注销“一网”服务。推行税务注销分类处理，大幅简化社保、商务、海关等注销手续，压缩企业注销时间。推进企业简易注销登记试点，对于被终止简易注销登记的企业，允许其符合条件后再次依程序申请简易注销。（市场监管总局牵头，人力资源社会保障部、商务部、海关总署、税务总局等国务院相关部门及各地区按职责分工负责）
　　2.推动各地研究建立办理破产工作统一协调机制，统筹推进破产程序中的业务协调、信息共享等工作。研究建立自然人破产制度，重点解决企业破产产生的自然人连带责任担保债务问题。（发展改革委牵头，国务院相关部门及各地区按职责分工负责）
　　（九）全面开展工程建设项目审批制度改革，压减审批时间和环节，在确保实现将审批时间压减到120个工作日以内的基础上，有条件的地方要进一步压减审批时间。（住房城乡建设部牵头，国务院相关部门及各地区按职责分工负责）
　　具体措施：
　　1.继续开展工程建设项目审批制度全流程、全覆盖改革，大力精简工程建设项目审批环节和事项，分类优化审批流程，实施联合审图和联合验收等。在试点地区探索取消施工图审查（或缩小审查范围）、实行告知承诺制和设计人员终身负责制，并加强事中事后监管。2019年底前在东部沿海地区率先实现“互联网＋工程审批”，在全国基本实现“一窗受理、并联办理”。（住房城乡建设部牵头，国务院相关部门及各地区按职责分工负责）
　　2.推进环评制度改革，对不涉及有毒有害及危险品的仓储、物流配送等一批基本不产生生态环境影响的项目，统一不再纳入环评管理。试点对环境影响小、风险可控的项目，简化环评手续或纳入环境影响登记表备案管理，2019年底前将项目环评审批时间压缩至法定时限的一半。（生态环境部牵头，国务院相关部门及各地区按职责分工负责）
　　3.推进用地规划“多审合一、多证合一”改革，合并用地预审和规划选址，整合建设用地规划许可证、建设用地批准书、国有土地划拨决定书，2019年底前减少用地规划相关证照，压缩办理时限。（自然资源部及各地区按职责分工负责）
　　（十）治理各种不合理收费，防止地方非税收入非正常增长、抵消减税降费的政策效果，决不能再增加收费项目，确保减税降费的目标落实到位。（财政部、发展改革委、民政部、税务总局、市场监管总局等国务院相关部门及各地区按职责分工负责）
　　具体措施：
　　1.2019年9月底前组织开展减税降费政策实施效果监督检查工作，重点关注非税收入增速异常和乱收费等情况，坚决纠正违规开征、多征、预征非税收入的行为，坚决查处乱收费、乱罚款和各种摊派等问题。（财政部负责）
　　2.治理政府部门下属单位、行业协会商会、中介机构等涉企收费行为，坚决清理整治乱收费和第三方截留减税降费红利行为。动态调整行政事业性收费和实行[政府定价的经营服务性收费目录清单](https://www.pkulaw.com/chl/73d03ec81307869abdfb.html)，全面公示收费项目、标准和依据。2019年9月底前制定出台进一步加强和改进行业协会商会收费管理的有关文件。（发展改革委、市场监管总局、工业和信息化部、民政部、财政部等国务院相关部门按职责分工负责）
　　3.降低企业物流成本，督促各地做好口岸收费目录清单公示，整治港口、船公司、物流堆场、货代、船代等乱收费、不合理收费行为，2020年底前将全国单个集装箱进出口环节常规收费压减至400美元以内。清理铁路、水路的货运和客运杂费，降低收费标准，2019年底前公布收费和处罚事项清单。（交通运输部、海关总署、发展改革委、财政部、市场监管总局、铁路局等国务院相关部门及各地区按职责分工负责）
　　4.2019年底前开展降低融资收费专项清理行动，规范中小企业融资时强制要求办理的担保、保险、评估、公证等事项，减少融资过程中的附加费用。（银保监会牵头，国务院相关部门按职责分工负责）

　　**二、**加强公正监管，切实管出公平
　　（十一）有关部门要分领域抓紧制订全国统一、简明易行的监管规则和标准，并向全社会公开。抓紧清理规范和修订完善那些边界宽泛、执行弹性大的监管规则和标准。（国务院相关部门按职责分工负责）
　　具体措施：
　　1.2019年底前对已取消下放的行政许可事项事中事后监管措施落实情况进行全面评估，按照法律规定和“三定”规定确定的监管职责，进一步明确相应的事中事后监管措施，确保监管全覆盖。（各地区、各部门负责）
　　2.2019年9月底前制定出台加强和规范事中事后监管的指导意见，落实放管结合、并重的要求，建立健全适合我国高质量发展要求、全覆盖、保障安全的事中事后监管制度。（国务院办公厅、市场监管总局负责）
　　3.2019年底前对现有的主要监管规则标准进行修订完善，尽可能消除模糊和兜底条款，并依法依规向社会公开。在市场监管、生态环境保护、交通运输、农业农村、文化市场等行政执法领域分别制定规范执法自由裁量权的办法，明确法律依据和处罚标准。（生态环境部、交通运输部、农业农村部、文化和旅游部、市场监管总局等国务院相关部门及各地区按职责分工负责）
　　4.2019年底前对现有涉企现场检查事项进行全面梳理论证，通过取消、整合、转为非现场检查等方式，压减重复或不必要检查事项，着力解决涉企现场检查事项多、频次高、随意检查等问题。持续清理规范行政处罚事项，对重复处罚、标准不一、上位法已作调整的事项及时进行精简和规范。（国务院相关部门及各地区按职责分工负责）
　　5.全面落实行政执法公示、执法全过程记录、重大执法决定法制审核三项制度，2020年底前基本实现各级行政执法机关及时准确公示执法信息、执法全过程留痕和可回溯管理、重大执法决定法制审核全覆盖。（司法部牵头，国务院相关部门及各地区按职责分工负责）
　　（十二）完善“双随机、一公开”监管制度和工作机制，推动日常监管“双随机、一公开”全覆盖。（市场监管总局牵头，国务院相关部门及各地区按职责分工负责）
　　具体措施：
　　推进“双随机、一公开”跨部门联合监管，以省为单位建立统一的监管平台，实现各部门监管信息与省级平台互联互通。2019年底前市场监管部门完成双随机抽查全流程整合，实现双随机抽查覆盖企业比例达5%。2020年底前在市场监管领域实现相关部门“双随机、一公开”监管全覆盖，地方各级人民政府相关部门联合“双随机、一公开”监管常态化。（市场监管总局牵头，国务院相关部门及各地区按职责分工负责）
　　（十三）对重点领域进行重点监管，特别是对疫苗、药品、特种设备、危险化学品等涉及到人民生命安全、社会关注度高的领域，要实行全主体、全品种、全链条严格监管。（应急部、市场监管总局、药监局等国务院相关部门及各地区按职责分工负责）
　　具体措施：
　　1.加快研究建立违法严惩制度、惩罚性赔偿和巨额罚款制度、内部举报人奖励制度等，提高重点监管效能，让严重违法者付出高昂成本。（市场监管总局牵头，国务院相关部门按职责分工负责）
　　2.2019年11月底前组织开展危险化学品质量安全风险隐患排查和专项整治。（市场监管总局等国务院相关部门及各地区按职责分工负责）
　　3.建立疫苗生产企业巡查检查制度，实施对全部在产疫苗生产企业全覆盖检查。推行向疫苗生产企业派驻检查员制度。开展中药饮片质量专项整治。加强对国家组织药品集中采购和使用试点中标品种的日常监管、产品抽检和不良反应监测，督促企业落实主体责任。（药监局牵头，国务院相关部门及各地区按职责分工负责）
　　4.开展打击欺诈骗保专项治理活动，实现对全国定点医疗机构和零售药店监督检查全覆盖，公开曝光欺诈骗保典型案例。（医保局负责）
　　（十四）加强社会信用体系建设，大力推进信用监管，推行承诺制，让市场主体和公民讲诚信，自主承诺。对违背承诺、搞虚假承诺甚至坑蒙拐骗的，一经发现要严厉惩罚。（发展改革委、人民银行、市场监管总局牵头，国务院相关部门及各地区按职责分工负责）
　　具体措施：
　　1.规范认定并设立市场主体信用“黑名单”，建立健全信用修复、异议申诉等机制。2019年底前修订《[严重违法失信企业名单管理暂行办法](https://www.pkulaw.com/chl/1fae27f4d1a0a8b1bdfb.html)》，完善经营异常名录和严重违法失信企业名单管理等制度。（发展改革委、市场监管总局等国务院相关部门按职责分工负责）
　　2.围绕信用承诺、信用修复、失信联合惩戒、信用大数据开发利用等工作，2020年启动信用建设和信用监管试点示范。（发展改革委牵头，各地区、各部门负责）
　　3.2020年底前建立全国统一的信用报告标准，推动信用报告结果实现异地互认。（发展改革委、人民银行牵头，各地区、各部门负责）
　　4.依托国家“互联网＋监管”等系统，有效整合公共信用信息、市场信用信息、投诉举报信息和互联网及第三方相关信息，充分运用大数据、人工智能等新一代信息技术，加快实现信用监管数据可比对、过程可追溯、问题可监测。（国务院办公厅、发展改革委、市场监管总局牵头，各部门按职责分工负责）
　　5.建立完善以信用为基础的新型海关监管机制，根据企业信用等级实施差别化通关监管措施，2019年底前出台对跨境电子商务等企业的认证标准。（海关总署负责）
　　6.推进知识产权领域信用体系建设，研究制定知识产权（专利）领域严重失信联合惩戒对象名单管理办法。加强对商标抢注和恶意注册、非正常专利申请等行为的信用监管。研究制定规范商标注册申请行为的有关规定。（知识产权局负责）
　　（十五）加快推进“互联网＋监管”，及时总结推广地方好的经验做法，提高监管及时性、精准性、有效性。（国务院办公厅牵头，国务院相关部门及各地区按职责分工负责）
　　具体措施：
　　1.2019年9月底前完成国家“互联网＋监管”系统主体功能建设并上线运行，归集共享各类监管数据，建立完善相关风险预警模型，实现对违法行为早发现、早提醒、早处置。（国务院办公厅负责）
　　2.加快推进地方和部门“互联网＋监管”系统建设并与国家“互联网＋监管”系统对接联通，推动形成统一规范、信息共享、协同联动的全国“互联网＋监管”体系。（国务院办公厅牵头，各地区、各部门负责）
　　（十六）坚持对新兴产业实施包容审慎监管，在监管中找到新生事物发展规律，该处置的处置，该客观对待的客观对待，不简单封杀，但也决不能放任不管，推动新业态更好更健康发展。（国务院相关部门及各地区按职责分工负责）
　　具体措施：
　　1.2019年8月底前出台[促进平台经济规范健康发展的指导意见](https://www.pkulaw.com/chl/783b288ab21b7449bdfb.html)，加大政策支持力度，创新监管方式，落实和完善包容审慎监管要求，推动建立健全适应平台经济发展特点的新型监管机制。（国务院办公厅牵头，国务院相关部门及各地区按职责分工负责）
　　2.优化新业态发展环境，放宽新兴行业企业名称登记限制，完善互联网平台企业用工、灵活就业人员相关政策，加强政府部门与互联网平台数据共享，2019年底前建成全国一体化在线政务服务平台电子证照共享服务系统。鼓励各地探索适应新业态特点、有利于公平竞争的公正监管办法，更好支持新业态发展。（国务院办公厅、市场监管总局、人力资源社会保障部等国务院相关部门及各地区按职责分工负责）

　　**三、**大力优化政府服务，努力服出便利
　　（十七）切实增强服务意识，不断提升服务能力和水平，大力提升政务服务效率，针对群众办事来回跑、环节多材料多、政府服务效率低等问题，对政务服务流程、方式进行系统化改革。（国务院办公厅牵头，国务院相关部门及各地区按职责分工负责）
　　具体措施：
　　1.2019年9月底前制定出台建立政务服务“好差评”制度有关指导意见，倒逼各级政府部门不断改进工作。（国务院办公厅负责）
　　2.加快实现公安、市场监管、住房城乡建设、税务、司法、民政等部门信息与不动产登记信息共享集成。对登记中涉及多个部门交叉办理的事项，通过信息化手段整合集成业务流程，2019年底前在全国基本实现“一窗受理、并行办理”，东部沿海地区力争率先实现办理一般登记、抵押登记时间压缩至5个工作日以内；2020年底前“互联网＋不动产登记”在地级及以上城市全面实施，办理一般登记、抵押登记时间力争全部压缩至5个工作日以内。（自然资源部牵头，国务院相关部门及各地区按职责分工负责）
　　3.提升跨境贸易便利化水平，加强国际贸易“单一窗口”与银行、保险、民航、铁路、港口等相关行业机构合作对接，2019年底前实现主要申报业务应用率达100%。进一步精简进出口环节监管证件数量，加快推行进出口“提前申报”、“两步申报”通关模式和无纸化通关作业。（海关总署牵头，交通运输部、银保监会、民航局、铁路局等国务院相关部门及各地区按职责分工负责）
　　4.试点建立统一的现代动产担保系统，2020年底前力争整合各类动产登记和权利担保登记系统，实现企业担保在一个平台上登记，各相关部门按职责分别实施后台监管。（人民银行牵头，交通运输部、市场监管总局、知识产权局等国务院相关部门按职责分工负责）
　　5.扩大异地就医结算范围，2019年底前基本实现异地就医患者在定点医院住院持卡看病、即时结算，2020年底前基本实现符合条件的跨省异地就医患者在所有定点医院能直接结算。（医保局及各地区按职责分工负责）
　　6.加快建成全国统一的社会保险公共服务平台，实现个人权益记录查询、自主认证、养老保险待遇测算、社保卡应用状态查询等“一站式”功能，全面取消领取社保待遇资格集中认证，优化社保卡服务，加快推进电子社保卡。2019年底前实现养老保险关系转移接续业务网上办理。（人力资源社会保障部及各地区按职责分工负责）
　　7.推广商标和专利电子化申请，2019年底前将高价值专利审查周期压缩至17.5个月、商标注册平均审查周期压缩到5个月，消减发明专利审查积压10万件。（知识产权局负责）
　　8.2019年10月底前改进优化来华工作外国人入境和居留管理制度办法，优化外国人来华相关审批、审查服务，压减办理时间。同时，向全社会开放出入境证件身份认证服务平台，为境内港澳居民、华侨持用出入境证件办理金融、教育、医疗等社会事务提供便利。（公安部、科技部、人力资源社会保障部、移民局按职责分工负责）
　　9.2020年6月底前全面推广证明事项告知承诺制。进一步改进和规范基层群众性自治组织出具证明工作，解决群众反映强烈的“万能居委会”、“社区万能章”等问题。（司法部、民政部按职责分工负责）
　　（十八）依托全国一体化在线政务服务平台，加快打造全国政务服务“一张网”，实现更大范围“一网通办”、异地可办、“掌上可办”，确需到现场办的再到政务服务大厅办理。在办理政务服务事项过程中要注意保护商业秘密和个人隐私。（国务院办公厅牵头，国务院相关部门及各地区按职责分工负责）
　　具体措施：
　　1.2019年9月底前上线运行以国家政务服务平台为总枢纽的全国一体化在线政务服务平台，建立完善全国一体化在线政务服务平台标准规范体系、安全保障体系和法规制度体系，在更大范围实现“一网通办”、异地可办。2019年底前首批推动10种高频电子证照全国标准化应用和互信互认，推动100项以上政务服务事项在各省（区、市）内和跨省（区、市）实现“一网通办”。2020年底前基本完成对依申请办理的政务服务事项名称、编码、依据、类型等基本要素和办事指南要素在国家、省、市、县四级统一。（国务院办公厅牵头，各地区、各部门负责）
　　2.更大力度推动央地数据共享，建立权威高效的数据共享协调机制，2019年再新增拓展1000项共享数据，将直接关系到企业和群众办事、应用频次高的数据优先纳入共享范围，满足30项以上全国普遍性高频政务服务事项的数据共享需求。加快推进“互联网＋可信身份认证”平台、全国人口信息社会应用平台等多种网上身份认证能力融合，为全国一体化在线政务服务平台提供统一身份认证支撑。（国务院办公厅、发展改革委、公安部按职责分工负责）
　　3.提升政务服务大厅“一站式”功能，2019年底前政务服务事项进驻综合性实体政务服务大厅基本实现“应进必进”，70%以上政务服务事项实现“一窗”分类受理。推动政务服务大厅与政务服务平台全面对接融合。（国务院办公厅牵头，各地区、各部门负责）
　　（十九）一些带有垄断性质的供电、供水、供气、供暖等公用事业单位及医院、银行等服务机构，要从方便市场主体和人民群众出发，提高服务质量和效率，大幅压减自来水、电力、燃气、供暖办理时间，提高相关政策透明度，大力推行APP办事、移动支付等。（发展改革委、住房城乡建设部、水利部、卫生健康委、人民银行、银保监会、能源局等国务院相关部门及各地区按职责分工负责）
　　具体措施：
　　1.2019年底前研究制定规范水电气暖等行业收费、管理、服务等方面的综合性意见。（发展改革委牵头，住房城乡建设部等国务院相关部门按职责分工负责）
　　2.进一步压减办电时间，加快推广低压小微企业用电报装“三零”（零上门、零审批、零投资）服务，2019年底前在全国各直辖市、省会城市实现低压小微企业用电报装“三零”服务，将办电时间压缩至30个工作日以内。地方政府要大幅压缩电力接入工程审批时间。研究建立针对供电企业停电超过一定频次和时间的处罚机制。（能源局及各地区按职责分工负责）
　　3.优化水气报装服务，指导地方落实新修订的《城镇供水服务》、《燃气服务导则》等国家标准，将水气设施报装提前到施工许可证核发后即可办理，2019年底前将供水新增、扩容改装的报装时间分别压缩至20个、15个工作日以内，将燃气报装时间压缩至16个工作日以内，大幅压减报装、安装费用。（住房城乡建设部、发展改革委及各地区按职责分工负责）
　　4.指导督促商业银行优化服务，2019年底前解决银行卡解绑和异地注销难、“睡眠卡”收费不透明等一批群众反映强烈的问题，加快推动解决继承财产支取难问题。优化电信服务，持续推进降低电信收费、异地销户、携号转网等工作。持续优化老年证、居住证、流动人员人事档案管理等证件或手续办理流程，减少公用企事业单位索要的证明材料。（银保监会、工业和信息化部、公安部、民政部、卫生健康委、人力资源社会保障部、发展改革委、住房城乡建设部、司法部等国务院相关部门及各地区按职责分工负责）
　　（二十）大力发展服务业，采用政府和市场多元化投入的方式，引导鼓励更多社会资本进入服务业，扩大服务业对外开放，结合城镇老旧小区改造，大力发展养老、托幼、家政和“互联网＋教育”、“互联网＋医疗”等服务，有效增加公共服务供给、提高供给质量，更好满足人民群众需求。（发展改革委、教育部、民政部、住房城乡建设部、商务部、卫生健康委等国务院相关部门及各地区按职责分工负责）
　　具体措施：
　　1.2019年8月底前制定出台加快发展“互联网＋社会服务”的意见，提出进一步推进“互联网＋”与教育、健康医疗、养老、文化、旅游、体育、家政等领域深度融合发展的政策措施，充分发挥社会领域公共服务资源作用，创新服务模式，更好惠及人民群众。（发展改革委牵头，教育部、民政部、商务部、文化和旅游部、卫生健康委、体育总局等国务院相关部门按职责分工负责）
　　2.结合城镇老旧小区改造，深入落实养老、托幼、家政等服务业有关政策意见。推进建设城乡便民消费服务中心，进一步扩大建设范围和数量，鼓励建设社区生活服务中心，在城乡社区推动包括家政服务在内的居民生活服务业发展，更好满足群众需要。支持社会力量发展普惠性托育服务，增加普惠性托育服务有效供给，促进婴幼儿照护服务标准化、规范化发展。（住房城乡建设部、商务部、民政部、卫生健康委、发展改革委等国务院相关部门按职责分工负责）
　　3.2020年底前落实互联网诊疗和互联网医院管理相关政策，推动二级以上医院普遍提供分时段预约诊疗、诊间结算、移动支付等服务。探索建立老年人长期护理需求认定和等级评定标准体系，加强医疗护理员培训，促进老龄健康服务业发展。（卫生健康委、医保局按职责分工负责）

　　**四、**强化责任担当，确保“放管服”改革不断取得新成效
　　（二十一）抓紧研究制定《优化营商环境条例》，为营商环境建设提供法治保障。2019年要开展营商环境百项诉求处理行动，破解一批营商环境痛点难点。（发展改革委、司法部、国务院办公厅牵头，国务院相关部门及各地区按职责分工负责）
　　具体措施：
　　1.抓紧研究制定《优化营商环境条例》，2019年8月底前完成向各有关方面征求意见和法规审查工作、形成草案，9月底前公布实施。（发展改革委、司法部、国务院办公厅牵头，国务院相关部门按职责分工负责）
　　2.围绕开办企业、办理建筑许可、获得电力、登记财产、纳税、跨境贸易等优化营商环境重点领域，搜集一批企业和群众反映强烈的问题，2019年底前推动各地区、各部门研究出台具体解决措施。（发展改革委牵头，国务院相关部门及各地区按职责分工负责）
　　3.对标国际先进水平，聚焦营商环境关键领域和薄弱环节，研究制定优化营商环境重点任务台账，并抓好落实。（财政部、国务院办公厅牵头，国务院相关部门及各地区按职责分工负责）
　　（二十二）鼓励支持各地大胆创新，及时指导帮助地方解决改革中遇到的难题。要进一步加大向地方放权特别是综合授权的力度，充分调动和发挥地方推进改革发展的积极性、主动性和创造性。（国务院相关部门及各地区按职责分工负责）
　　（二十三）对滞后于改革要求、不利于优化营商环境、制约新产业新业态新模式发展的有关规定，要加快清理修改。对与改革决策相抵触的行政法规、部门规章和行政规范性文件，要应改尽改、应废尽废。涉及到修改法律的，要与改革方案同步提出修法建议。各地区可通过制定地方性法规、规章，将实践证明行之有效、人民群众满意、市场主体支持的改革举措固化下来。（各地区、各部门负责）
　　（二十四）要把“放管服”改革、优化营商环境各项举措落实情况，作为2019年国务院大督查的重点。继续用好督查奖惩这个有效办法，对成效明显的加大表扬和政策激励力度，对不作为乱作为延误改革的要严肃问责。（国务院办公厅牵头，各地区、各部门负责）
　　各地区、各部门的贯彻落实情况，年底前书面报国务院。工作中取得的重大进展、存在的突出问题要及时报告。

© 北大法宝：（[www.pkulaw.com](https://www.pkulaw.com/)) 专业提供法律信息、法学知识和法律软件领域各类解决方案。北大法宝为您提供丰富的参考资料，正式引用法规条文时请与标准文本核对。
欢迎查看所有[产品和服务](https://www.pkulaw.com/helps/2.html)。法宝快讯：[如何快速找到您需要的检索结果？](https://www.pkulaw.com/helps/70.html)[法宝](https://www.pkulaw.com/helps/69.html)V6有何新特色？

扫描二维码阅读原文

**原文链接：**<https://www.pkulaw.com/chl/1de6dafed1dae1febdfb.html>
